# Potential bias and lack of generalizability in electronic health record data: reflections on health equity from the National Institutes of Health Pragmatic Trials Collaboratory

**DOI:** 10.1093/jamia/ocad115

**Published:** 2023-06-26

**Authors:** Andrew D Boyd, Rosa Gonzalez-Guarda, Katharine Lawrence, Crystal L Patil, Miriam O Ezenwa, Emily C O’Brien, Hyung Paek, Jordan M Braciszewski, Oluwaseun Adeyemi, Allison M Cuthel, Juanita E Darby, Christina K Zigler, P Michael Ho, Keturah R Faurot, Karen L Staman, Jonathan W Leigh, Dana L Dailey, Andrea Cheville, Guilherme Del Fiol, Mitchell R Knisely, Corita R Grudzen, Keith Marsolo, Rachel L Richesson, Judith M Schlaeger

**Affiliations:** Department of Biomedical and Health Information Sciences, University of Illinois Chicago, Chicago, Illinois, USA; Duke University School of Nursing, Durham, North Carolina, USA; Department of Population Health, New York University Grossman School of Medicine, New York City, New York, USA; College of Nursing, University of Illinois Chicago, Chicago, Illinois, USA; University of Florida College of Nursing, Gainesville, Florida, USA; Department of Population Health Sciences, Duke University School of Medicine, Durham, North Carolina, USA; Biostatistics (Health Informatics), Yale University, New Haven, Connecticut, USA; Henry Ford Health, Detroit, Michigan; Ronald O. Perelman Department of Emergency Medicine, New York University Grossman School of Medicine, New York City, New York, USA; Ronald O. Perelman Department of Emergency Medicine, New York University Grossman School of Medicine, New York City, New York, USA; College of Nursing, University of Illinois Chicago, Chicago, Illinois, USA; Duke University School of Medicine, Durham, North Carolina, USA; Division of Cardiology, University of Colorado School of Medicine, Aurora, Colorado, USA; Department of Physical Medicine and Rehabilitation, University of North Carolina School of Medicine, Chapel Hill, North Carolina, USA; Duke University School of Medicine, Durham, North Carolina, USA; College of Nursing, University of Illinois Chicago, Chicago, Illinois, USA; Physical Therapy, St. Ambrose University, Davenport, Iowa, USA; Department of Physical Therapy and Rehabilitation Science Department, University of Iowa, Iowa City, Iowa, USA; Mayo Clinic Comprehensive Cancer Center, Rochester, Minnesota, USA; Department of Biomedical Informatics, University of Utah School of Medicine, Salt Lake City, Utah, USA; Duke University School of Nursing, Durham, North Carolina, USA; Department of Medicine, Memorial Sloan Kettering Cancer Center, New York City, New York, USA; Department of Population Health Sciences, Duke University School of Medicine, Durham, North Carolina, USA; Department of Learning Health Sciences, University of Michigan Medical School, Ann Arbor, Michigan, USA; College of Nursing, University of Illinois Chicago, Chicago, Illinois, USA

**Keywords:** health equity, patient-reported outcomes, social determinants of health, community engagement, health literacy

## Abstract

Embedded pragmatic clinical trials (ePCTs) play a vital role in addressing current population health problems, and their use of electronic health record (EHR) systems promises efficiencies that will increase the speed and volume of relevant and generalizable research. However, as the number of ePCTs using EHR-derived data grows, so does the risk that research will become more vulnerable to biases due to differences in data capture and access to care for different subsets of the population, thereby propagating inequities in health and the healthcare system. We identify 3 challenges—incomplete and variable capture of data on social determinants of health, lack of representation of vulnerable populations that do not access or receive treatment, and data loss due to variable use of technology—that exacerbate bias when working with EHR data and offer recommendations and examples of ways to actively mitigate bias.

## INTRODUCTION

Efforts to minimize current knowledge gaps regarding the effectiveness of healthcare interventions in real-world contexts have propelled the use of embedded pragmatic clinical trials (ePCTs). In traditional explanatory clinical trials, the goal is to test efficacy of an intervention. Research is conducted separate from care, with dedicated staff and specific mechanisms for recruiting participants and collecting data. In contrast, with ePCTs, typical patients at clinical sites are randomized to treatment arms, and research is conducted in real-time as part of routine care leveraging existing clinicians, staff, and workflows. In addition, the interventions in ePCTs often rely on electronic health record (EHR) systems to facilitate the implementation and evaluation of both usual care and interventions.^1^ EHR data are used for feasibility assessments, study screening and enrollment, baseline data collection, tailoring interventions, implementation, and outcome assessments.^2^^,^^3^

Despite great promise, the value of evidence from the EHR is limited because of threats to internal validity due to biased results and to external validity due to poor generalizability. Biased results and poor generalizability can occur because detailed information about specific populations is missing, and critically, is missing not at random: these data are disproportionately missing in diverse and underserved populations.^4^

The NIH Pragmatic Trials Collaboratory supports the design, conduct, and dissemination of large-scale ePCTs that address significant public health challenges. Many in this research community have encountered challenges, including lack of representation of diverse and underserved populations that experience disparities in access to care, incomplete and variable capture of SDOH data, and data loss due to variable use of technology when working with EHR data. For this article, the EHR, Scientific Equity and Diversity, and Patient-Centered Outcomes Core working groups from the NIH Pragmatic Trials Collaboratory share experiences leveraging EHRs for ePCTs and offer examples of their solutions for addressing the challenges (Table 1).

**Table 1.** Case examples from the NIH Pragmatic Trials Collaboratory

Trial and goal	Solution	Recommendation
Challenge 1: Engagement, recruitment, and follow-up of diverse and underserved populations		
BeatPain Utah^5^ This trial compares the effectiveness of nonpharmacologic intervention strategies for patients with chronic low back pain seeking care in Federally Qualified Health Centers (FQHCs) throughout the state of Utah.	Utah CHC patients are 50% Hispanic, 8% Native American, 38% best served in a language other than English, 61% are below 100% of Federal Poverty Level, 45% are uninsured, and 41% of the clinics are in rural areas (RUCC >4). The trial uses electronic referrals within the EHR and text message-based patient engagement strategies that maximize reach for recruitment, intervention delivery, and retention. The trial also uses culturally adapted text messaging for patient engagement, with messages delivered in English or Spanish according based on the patient’s language preference documented in the EHR.	When planning ePCTs, sponsors and investigators can intentionally engage with a variety of settings serving diverse and underserved populations and uses various recruitment strategies and culturally adapted engagement to help improve diversity and representation.
Challenge 2: Lack of access to complementary and integrative health services		
GRACE^6^ This trial evaluates the effects of guided relaxation and acupuncture for people with sickle cell disease, the majority of whom identify as Black.	To address the structural barriers to both CIH and CIH-related research inclusion for underserved communities, the GRACE trial is expanding the range of community locations for acupuncture and is providing transportation coverage, tablets, and data plans if needed. To ensure consistent capture of both demographics and SDOH data from PROs, this trial linked the EHR data to REDCap.	Insurance coverage, transportation, and technology barriers are contributing factors that produce access inequities and can be considered when planning a trial. For the collection of patient-reported outcomes such as SDOH, consider using technologies such as REDCap.
Challenge 3: Incomplete capture of important data on race, ethnicity, and SDOH		
Guiding Good Choices for Health^7^ This trial tests the feasibility and effectiveness of implementing a universal evidence-based anticipatory guidance curriculum (Guiding Good Choices) for parents of early adolescents to promote family bonds, healthy development, and reduce risky behaviors.	The surveys explicitly include comprehensive demographic (eg, races that are often not included in EHR data) and gender identity (trans, nonbinary, queer, questioning) questions. Self-report demographics may provide more accurate data than those originally reported to the health systems by parents or documented by providers. This is especially true for mixed race children, whose race or ethnicity may have been recorded at birth and reflect the race and/or ethnicity of the parent. Similarly, self-reported gender identity will supplement EHR data, which are rarely and suboptimally collected in health systems, in general and especially for this age group. These data could be used, for example, to examine potential disparities in the prevalence of mental health symptoms experienced by sexual minority youth during the pandemic.	Purposeful and systematic collection of both demographic and SDOH variables may help researchers to more thoroughly describe and understand study outcomes.
PRIM-ER^8^ This pragmatic, cluster-randomized stepped-wedge trial tests the effectiveness of Primary Palliative Care for Emergency Medicine (PRIM-ER) in 35 EDs across the United States. PRIM-ER is an education, training, and technical support quality improvement (QI) intervention with 4 core components: (1) evidence-based multidisciplinary primary palliative care education, (2) simulation-based workshops on communication in serious illness, (3) CDS, and (4) provider audit and feedback.	Aims to optimize the use of the EHR by creating a CDS tool to identify high risk patients most likely to benefit from primary palliative care and provide point-of-care clinical recommendations.	Intentionally extracting data on SDOH to improve the quality of EHR data may, therefore, require providers in inpatient and outpatient settings initiating focused discussions on specific SDOH. Intentionally alerting Emergency Medicine providers through a CDS tool to engage in goals of care conversations may serve as a subtle way to create the awareness of the need of capturing measures of SDOH and improve EHR data quality
Challenge 4: Lack of adoption of advanced EHR features in low-resource settings		
BeatPain Utah^5^ This trial compares the effectiveness of nonpharmacologic intervention strategies for patients with chronic low back pain seeking care in Federally Qualified Health Centers (FQHCs) throughout the state of Utah.	The research team has provided technical support to study sites to implement (1) electronic referrals within EHRs and (2) automated patient outreach via bidirectional text messaging to connect patients to telehealth-based low back pain management interventions at the University of Utah.	To counter the lack of adoption of advanced EHR features (eg, electronic referrals, patient portals) at low-resourced settings, providing additional support for sites can be helpful.
Challenge 3: Lack of technology and health literacy can reduce study enrollment, collection of important patient-reported outcomes, and effectiveness of study interventions among specific groups.^9^		
Nudge^10^^,^^11^ This trial aims to improve adherence to cardiovascular medications at health systems that serve historically marginalized patient groups.	The trial team tested the messages with patients and stakeholder groups to make sure the text messages were at a fifth grade level.	Ensuring that interventions are developed at the appropriate reading level is critical, as is ensuring that barriers to understanding and using interventions are minimized.
OPTIMUM^12^ This trial aims to determine the impact of a group-based mindfulness intervention for patients with chronic lower back pain.	The recruitment methods include mailing opt-out letters and flyers through the US postal service. Those who do not opt out are contacted by telephone, often prior to an upcoming office visit. In addition, flyers are embedded in the EHR that can be printed with post-visit instructions. These strategies have resulted in an ethnically diverse study population (only 47% White).	Employing multiple recruitment methods can help reach an underserved but potentially eligible population.
NOHARM^13^ This trial aims to encourage use of validated nonpharmacologic approaches to manage perioperative pain.	The team parameterized the EHR to direct outreach to nonportal using patients through printed mailings and telephone calls.	Granular EHR data regarding types and frequency of portal use can be leveraged to match outreach strategies with patients’ IT usage patterns.

### Challenge 1: Engagement, recruitment, and follow-up of diverse and underserved populations

Differences in access to healthcare persist across settings and populations, which impacts the presence and completeness of EHR data for diverse and underserved populations. Data in the EHR reflect the experience of those who have access to primary and outpatient care. Patients who are historically marginalized by the healthcare system (including those living in rural locations,^14^ undocumented,^15^ or underinsured and uninsured populations^15^) are less likely to seek primary and outpatient care and more likely to seek care in the emergency department (ED).^16^^,^^17^ Thus, the majority of clinical trial participants are White and have a higher socioeconomic status than the general population on average.^18–20^ As a result, racial/ethnic subgroup analyses are uncommon: as of 2019, only 13.4% of studies analyzed or reported outcomes by race and ethnicity.^21^ Also, populations from historically marginalized groups, with low socioeconomic status or those from rural areas, may also be underrepresented in the EHR. This absence of clinical care and potential absence of follow-up care, and resulting absence of data can lead to unequal outcome ascertainment and subsequent bias (ie, getting the wrong answer) in an ePCT.

To mitigate this problem, when planning ePCTs, sponsors and investigators from the NIH Collaboratory have engaged with a variety of settings serving diverse populations (eg, community health centers, local health departments, advocacy groups, and community health worker agencies) and use various recruitment strategies and culturally adapted engagement to help improve diversity and representation (Table 1).

### Challenge 2: Lack of access to complementary and integrative health services

Even among patients who are able to access healthcare, there are significant disparities in the types of services availed to them. For example, the positive impact of complementary integrative health (CIH) therapies on several health and chronic pain outcomes is well established,^22–24^ although few studies explicitly target people who are underserved or from racial and ethnic minority groups.^25^^,^^26^ Unequal access to CIH therapies due to insurance and transportation barriers leads to knowledge gaps about CIH therapy effectiveness more broadly.^27^ A subset of the NIH Pragmatic Trials Collaboratory focuses on CIH research explicitly includes people from racial and ethnic minority groups in an effort to address access inequities for these evidence-based strategies (Table 1).

### Challenge 3: Incomplete capture of important data on race, ethnicity, and social determinants of health

Data on race and ethnicity and social determinants of health (SDOH) are inconsistently and inaccurately captured in EHR systems as part of clinical care. SDOH data include information on safe housing, neighborhoods, transportation, educational and job opportunities, access to nutritious food, and physical activity; and are frequently missing (disproportionately by those with greater needs), which limits the ability to understand and address health inequities.^28^^,^^29^ While we lack a complete understanding of how SDOH factors may affect the etiology and natural history of disease, these data are critical for understanding the whole picture of patient care, population health, and systems care delivery. Lack of these data limits the ability to identify patients that might benefit from an intervention, the effectiveness of an intervention that might help patients overcome specific SDOH challenges, and the trialists’ ability to conduct careful subgroup analyses on social mediators and moderators. In a broader context, collection of these data can support health systems and providers to more appropriately classify patient complexity, identify appropriate interventions to meet various needs, and transform care with integrated services and community partnerships to improve health outcomes and reduce health inequities, while also saving costs.^30^ In the examples from the NIH Pragmatic Trials Collaboratory, strategies to overcome these barriers include the use of granular surveys and clinical decision support (CDS) tools to nudge providers to collect these data (Table 1).

### Challenge 4: Lack of adoption of advanced EHR features in low-resource settings

The incompleteness of key SDOH data is often coupled with a lack of adoption of advanced EHR features (eg, patient-friendly data collection interfaces including easy-to-use voice and text data collection tools, integration of data from other EHR systems and community and public health sources, EHR optimization services to enhance care coordination, and targeted reminders) that enable the capture of these data,^9^ particularly in low-resourced or busy clinical settings (eg, emergency care), which also impacts the representativeness and completeness of the EHR. Additionally, low-resourced healthcare settings like community health centers typically use a variety of EHR products that lack customization capabilities, and in our examples, trialists have provided additional support to low-resourced settings (Table 1).

### Challenge 5: Lack of technology and health literacy can reduce study enrollment, collection of important patient-reported outcomes, and effectiveness of study interventions among specific groups

Patients seeking care in low-resourced settings are less likely to use technology (eg, email or patient portals) or to provide their own previsit data through electronic approaches.^9^ When collected on paper forms, these data may not be completely entered into the EHR record or stored as scanned images. Issues of health literacy can be exacerbated by the introduction of health technologies—which require additional skills to navigate, incorporate, and interpret. These issues can reduce study enrollment, collection of important patient-reported outcomes, and effectiveness of study interventions among specific groups. Using patient-facing EHR modalities alone (ie, patient portals) for these tasks can limit access to potentially beneficial interventions for underserved groups due to their lower levels of adoption. To address these issues, our examples include understanding which patients use a portal, developing different strategies of outreach for those who do not use a portal, and ensuring an appropriate reading level and culturally sensitive presentation for materials (Table 1).

## CONCLUSION

ePCTs play a vital role in addressing current population health problems, and because of their use of EHR data and systems promises efficiencies that will increase the speed and, ultimately, the volume of relevant and generalizable research. However, as the number of ePCTs using EHR-derived data grows, so does the risk that research will become more vulnerable to biases due to differences in data capture and access to care for different subsets of the population, thereby propagating inequities in health and the healthcare system. Key to detecting and mitigating these biases is the implementation of approaches deliberately designed to ensure the inclusion and retention of patients, as well as the complete and accurate collection of data that can identify underrepresented populations and support measurement of health inequities.

The highlighted pragmatic trials (Table 1) are gathering evidence that is relevant to the unique aspects of different people’s health and offer examples for the routine and efficient collection of SDOH; these necessary analyses will illustrate the importance of doing so. By improving data capture, access to care, and patient technology support, ePCTs hold the potential to yield insights and estimates pertinent to the entire population, not just a subset of the population, and will set the stage for more systematic approaches.

## DISCLOSURES

ECO reports grants to her institution from Pfizer, BMS, and Novartis. KM reports grants and contracts to his institution from Novartis, Amgen, Seqirus, Genentech, BMS, and Boehringer Ingelheim. ADB reports grants from Alike Health, travel from Microsoft. All other authors have nothing to disclose.

## Data Availability

No data were generated as part of this manuscript.
